# Intraprosthetic Dislocation of Dual-Mobility Total Hip Arthroplasty: The Unforeseen Complication

**DOI:** 10.7759/cureus.19858

**Published:** 2021-11-24

**Authors:** Shady Hermena, Waleed Tawfeek, Paul Latimer

**Affiliations:** 1 Trauma and Orthopaedics, Yeovil District Hospital NHS Foundation Trust, Yeovil, GBR

**Keywords:** dual-mobility cup, dual-mobility total hip arthroplasty, dual-mobility bearings, intraprosthetic dislocation, dual mobility

## Abstract

Total hip arthroplasty (THA) is one of the most successful and widely accepted orthopedic procedures. Instability after THA is one of the most significant postoperative complications. Dual-mobility THA components were introduced in 1974 to overcome the risk of instability by increasing the jump distance. Dual-mobility bearings couple two articulations, namely, one between a 22-28 mm prosthetic head and polyethylene liner and another larger articulation between the polyethylene liner and the metal cup. Dislocation of the polyethylene liner and the consequent direct articulation between the prosthetic head and metal cup is recognized as intraprosthetic dislocation (IPD). This mode of THA failure is specific to dual-mobility implants. Despite the reduced incidence of IPD in modern dual-mobility implants compared to the early designs, iatrogenic IPD can occur during closed reduction of dislocated polyethylene liner-metal cup articulation. IPD requires timely diagnosis and early surgical intervention to minimize the necessity of major revision surgeries. This study presents a comprehensive review for dual-mobility-bearing THA, including the history and biomechanics, and focuses on the pathomechanics, diagnosis, and management of IPD.

## Introduction and background

Total hip arthroplasty (THA) is one of the most successful orthopedic procedures widely used to treat advanced hip joint arthritis and femoral neck fractures [1,2]. More than 450,000 THA procedures are performed in the United States every year, with expectations to reach 572,000 by 2030 [3]. In the United Kingdom, approximately 81,000 THA procedures are performed every year [4]. Instability of the prosthesis after THA is a serious complication with an incidence rate of 7% after primary THA and 25% after revision THA [3,5]. Risk factors for THA dislocation can be categorized into patient-related and operative procedure-related risk factors. Patient-related factors include a high body mass index, neurological disorders, previous spinal fusion surgery, THA performed for a neck of femur fracture, avascular necrosis, and rheumatoid arthritis [5-8]. Operative risk factors include the surgical approach, malposition of the components, small femoral head, and inadequate soft tissue tension [5,7,9-11]. Various operative techniques and strategies have been described to minimize the risk of dislocation after THA, for example, hip capsule and external rotator repair in the setting of posterior hip approach and maintaining the soft tissue tension [5]. Implant modifications to minimize the dislocation risk include utilizing larger femoral head diameter, constrained acetabular components, posteriorly raised acetabular liner, and dual-mobility acetabular cup. Dual-mobility-bearing implants have been widely used and shown to reduce the dislocation rate after primary and revision THA [12]. There are two articulations in a dual-mobility-bearing implant, namely, an inner articulation incorporating a capture mechanism between the fixed prosthetic head and polyethylene liner [13]. The unconstrained outer articulation is between the polyethylene liner and the metal shell. Because of the presence of two articulations, a specific mode of failure can affect dual-mobility THA due to the dislocation of the inner head from the polyethylene line. This mode of failure is known as intraprosthetic dislocation (IPD) [13]. IPD cannot be managed by closed reduction only and necessitates a revision of the polyethylene component [14]. The failure to identify this specific mode failure may result in acetabular cup damage due to direct articulation between the prosthetic femoral head and the highly polished interior cup surface [15]. This study presents a review of dual-mobility-bearing THA with a focus on IPD as a specific mode of failure in these implants. This review discusses the incidence, types, identification, and management of IPD.

## Review

History of dual-mobility components

The concept of THA dual-mobility cups was first introduced in France by Bousquet and Rambert in 1974 to overcome the risk of dislocation after THA [16,17]. The first dual-mobility cup was called the NOVAE, which incorporated two articulations to increase the jump distance and THA stability. In the NOVAE implant, the polyethylene liner was articulated with the highly polished internal surface of the acetabular cub. Moreover, the prosthetic head and the overlying polyethylene liner were designed to act as a large femoral head within the acetabular cup. Therefore, the NOVAE dual-mobility bearing implant combined Charnley’s low friction principle with the McKee-Farrar principle of the large femoral head [17,18].

The NOVAE acetabular cup was uncemented plasma-sprayed alumina coated with an inner stainless steel-polished surface [16,17]. The NOVAE incorporated a 22.2 mm metallic head to articulate a polyethylene liner. The polyethylene liner was made using ultra-high-molecular-weight polyethylene [12,18]. The NOVAE cup was designed to be press-fit fixed to the bony acetabulum via a three-point fixation system, including a 4.5 mm iliac screw and two Morse taper pegs [12]. Over the last four decades, dual-mobility implants have gone through many advancements, for example, the alumina coating was replaced by titanium and hydroxyapatite, the introduction of multiple screws to fix the metal shell, and a highly crosslinked ultra-high-molecular-weight polyethylene liner [12]. Various designs of cemented and uncemented dual-mobility implants are currently available in the market. Dual-mobility implants have been used in Europe for many years with successful outcomes; however, it has been only approved in the United States since 2009 [18]. The American Joint Replacement Registry data show a continuous increase in the use of dual-mobility-bearing THA for primary and revision hip arthroplasty procedures [19,20]. From 2012 to 2019, the usage of dual-mobility bearing for primary hip arthroplasties increased from 4.1% to 8.6% and from 14% to 22.3% for revision hip arthroplasties [20].

Biomechanics and design of dual-mobility implants

Dual-mobility-bearing THA incorporates a small prosthetic head (22 or 28 mm in diameter) which is freely mobile but constrained within a larger polyethylene liner [12,17,18]. The small prosthetic head is snap-fitted within the polyethylene liner [18]. The outer polyethylene liner, in turn, articulates with the highly polished inner surface of the metallic acetabular cup. Combining these two articulations within the dual-mobility-bearing THA enhances the range of movement and increases the jump distance compared to conventional THA [12]. The jump distance is the distance the femoral head center requires to move laterally prior to dislocation [21]. Increasing the jump distance lowers the risk of dislocation in THA [21]. In the dual-mobility setting, the prosthesis motion initiates in the smaller articulation (prosthetic head-polyethylene liner articulation) to the point where the prosthetic neck starts to impinge with the polyethylene liner rim. At this stage, THA movement occurs in the second articulation (polyethylene liner-metal cup articulation) [12]. An added theoretical advantage of dual mobility is that the head-liner complex acts as a larger head within the metal cup, increasing the head-neck ratio and increasing prosthesis stability [12].

Incidence of intraprosthetic dislocation

IPD is a specific complication of THA incorporating dual-mobility-bearing components [15-17]. The incidence of IPD was higher in early generations of dual-mobility THA implants, ranging from 2% to 4% [17,21-23]. More recent studies have shown a lower incidence of IPD of ranging 0-0.3% [24,25]. The modern modifications to dual-mobility components, designs, and sterilization techniques reduced the incidence of IPD [26]. The modern advances in the dual-mobility industry include using highly crosslinked ultra-high-molecular-weight polyethylene liners, liner-retentive rim modifications, and vacuum sterilization to reduce the free radical damage and hydroxyapatite-coated acetabular cups [26]. Iatrogenic IPD can occur during closed reduction of dislocated THA in the presence of dual-mobility acetabular components. One study reported an incidence of IPD of 71% of the included dual-mobility THA after trials of closed reduction for dislocated polyethylene liner-metal cup articulation [27].

Types and causes of intraprosthetic dislocation

IPD may not be proceeded by a traumatic event and can occur at any point after dual-mobility THA surgery. The leading cause of IPD is polyethylene liner wear, especially before introducing ultra-high-molecular-weight polyethylene liners [13]. In 2013, Philippot et al. published a classification system for IPD of dual-mobility components based on clinical and radiographic findings [26]. Their prospective study, which included 1,960 dual-mobility THAs, reported the intraoperative findings from 80 cases of IPDs [26]. Philippot et al. classified IPD into three main types. Type I was mainly caused by the wear of the retention rim of the polyethylene liner. In type I, the dual mobility movements of the prosthesis components were functioning freely without restriction. In type II, the blocked articulation between the polyethylene liner and the metal cup due to arthrofibrosis or heterotopic ossifications was the primary cause of IPD. However, in type III, IPD was induced by aseptic loosening of the acetabular cup [26].

**Figure 1:**
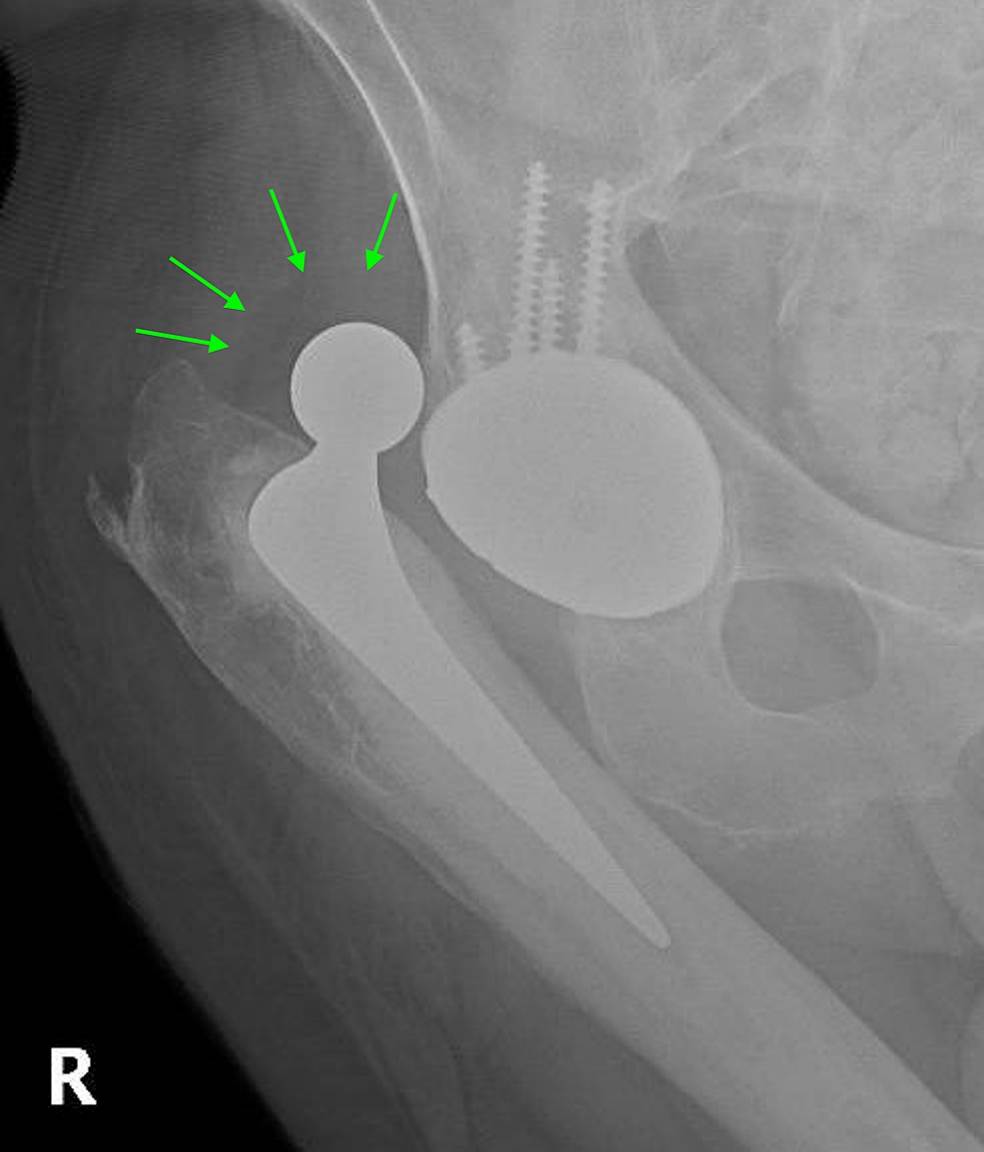
Anteroposterior X-ray of the right hip showing dislocated polyethylene liner-metal cup articulation of dual-mobility total hip arthroplasty. The polyethylene liner is attached to the prosthetic head, as demonstrated by the green arrows.

**Figure 2:**
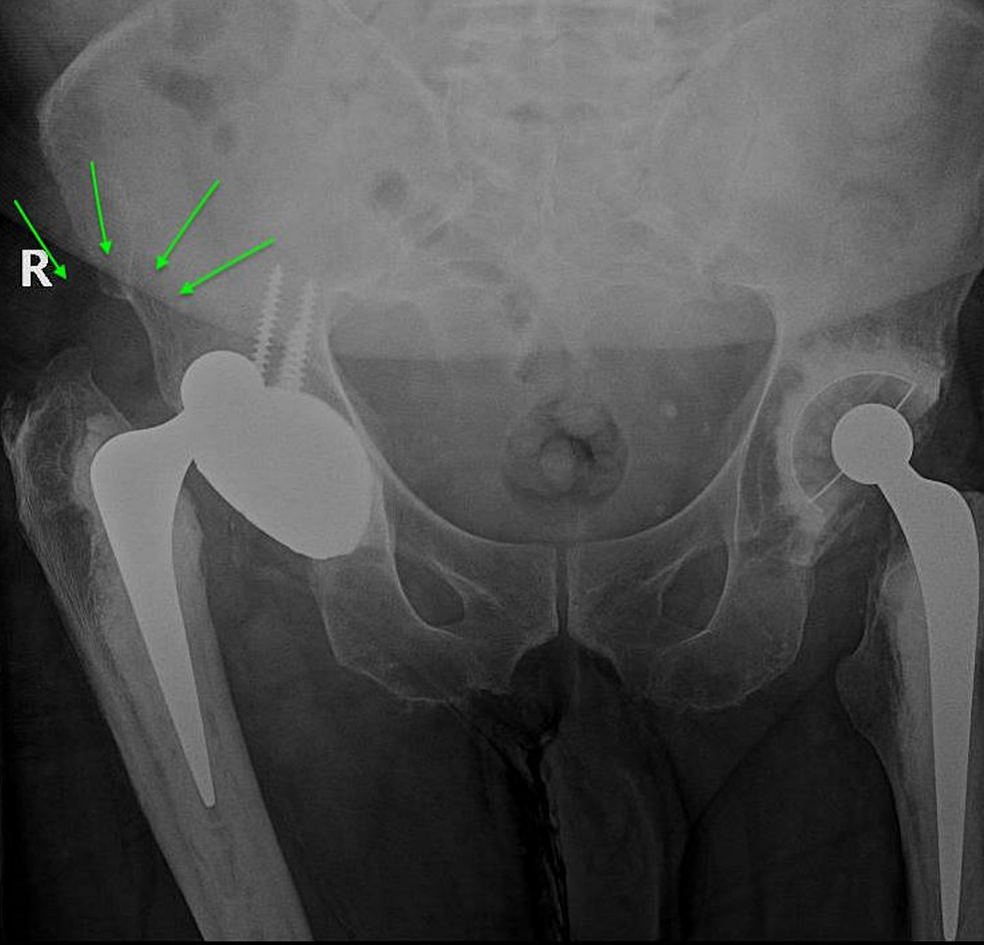
Anteroposterior pelvic X-ray showing intraprosthetic dislocation. The dislocated polyethylene liner is demonstrated by green arrows and appears as “the bubble sign.”

Iatrogenic IPD may occur without polyethylene liner wear during closed reduction of the polyethylene liner-metal cup dislocation (Figures 1, 2). The polyethylene liner might be caught at the edge of the metal cup or bony pelvic prominence during the manipulations causing dissociation of the prosthetic head-polyethylene liner capture mechanism, which is known as the bottle-opener effect [14]. Therefore, it is crucial to identify if the dislocated THA has dual-mobility-bearing components before attempting closed reduction. In such a setting, proper sedation and muscle relaxation or general anesthesia are required to minimize the force required and avoid excessive traction [28]. Moreover, it is recommended to perform the closed reduction of dual-mobility THA under fluoroscopic guidance without forceful levering which might cause the bottle-opener effect [14].

Presentation and diagnosis of intraprosthetic dislocation

Due to the direct articulation between the prosthetic head and the metal cup, limbing, leg shortening, and grinding sensations in the affected limb are common findings in IPD. Rarely, IPD may only present with hip discomfort and leg weakness [29].

**Figure 3:**
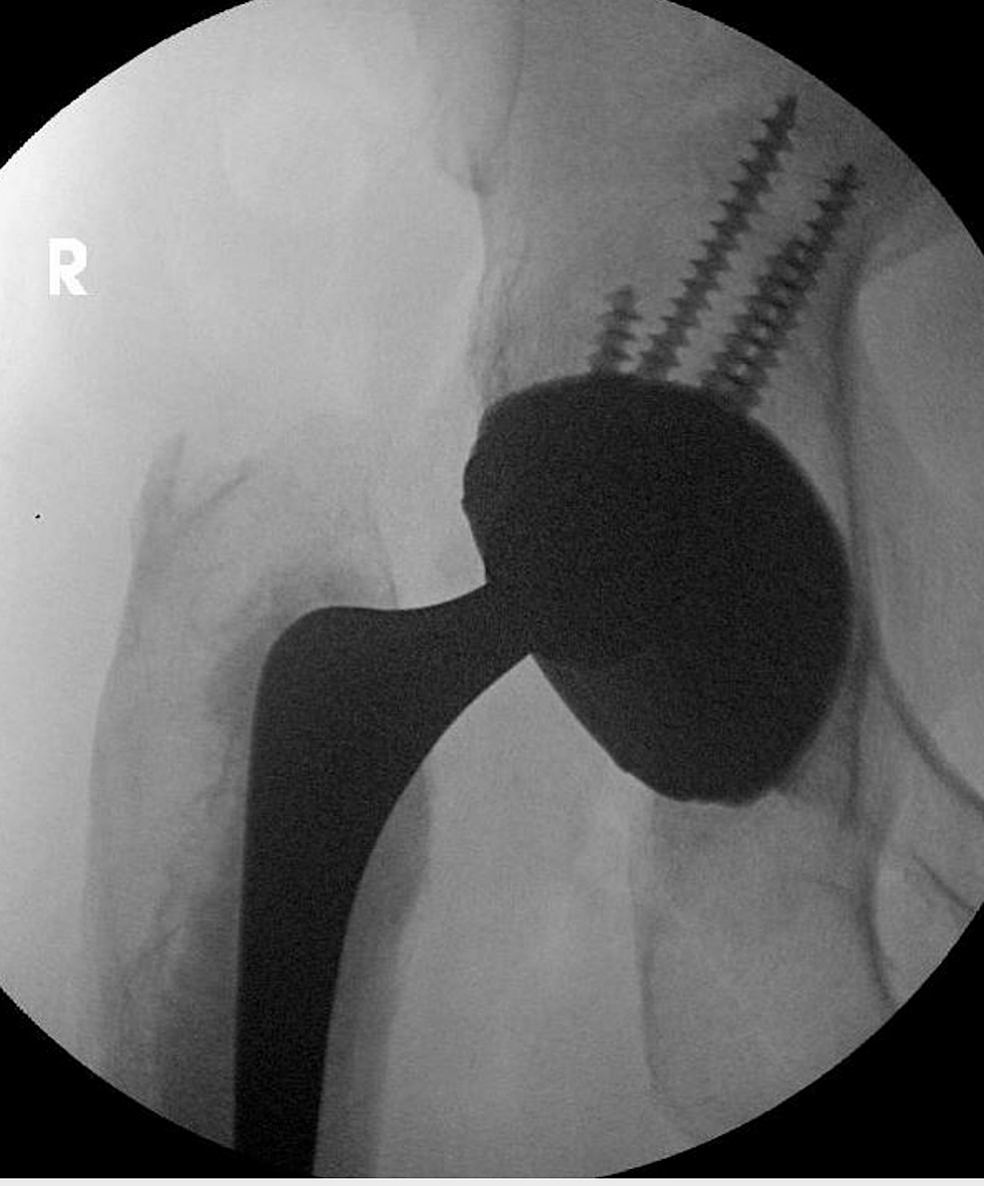
Fluoroscopy image of the right hip showing the eccentric position of the prosthetic head within the acetabular cup.

Plain radiographs can help identify an eccentric position of the prosthetic head within the cup due to the direct contact between the head and the metal cup (Figure 3). This sign may mimic the radiological presentation of polyethylene liner wear in a conventional THA. High suspicion of IPD should be considered if these X-rays were obtained for a dual-mobility-bearing implant, mainly if it was proceeded by a closed reduction maneuver. The dislocated polyethylene liner, despite being radiolucent, can be visualized outside the cup, which is known as the “bubble sign” (Figure 2) [14]. The outer polyethylene liner may migrate deep into the pelvis and may not be retrievable [30].

**Figure 4:**
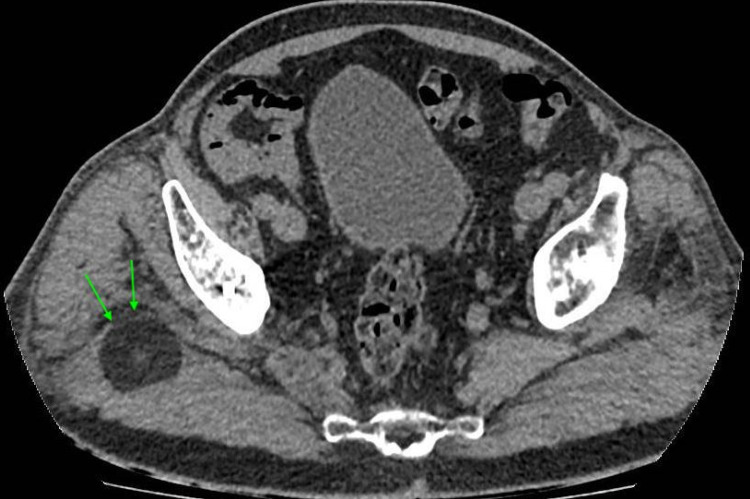
A cross-section image from a pelvic CT scan showing the dislocated polyethylene liner demonstrated by green arrows. CT: computed tomography

If the “bubble sign” is not clear or the plain radiographs are not conclusive, a computed tomography (CT) scan should be obtained [13]. CT scan can demonstrate the current position of the dislocated polyethylene liner (Figure 4) and provide more detailed information regarding the prosthesis components for better operative planning.

Management of intraprosthetic dislocation of dual-mobility implants

Metal cup in dual-mobility THA implants is not designed for direct articulation with the prosthetic head. The delay in IPD diagnosis may cause damage and wear of the prosthetic head and/or the acetabular metal cup. The friction between the prosthetic head and metal cup wear may result in significant soft tissue metallosis and raised cobalt and chromium levels [29,31]. Early identification of IPD may reduce the risk of major revision procedures.

IPD requires an operative intervention to revise the damaged polyethylene liner and restore dual-mobility articulations (prosthetic head-polyethylene liner and polyethylene liner-metal cup articulations). It is recommended to obtain a CT scan to assess the alignment of the acetabular cup and identify any prosthetic damage prior to the surgery. During the revision surgery, the acetabular metal cup should be checked for stability and signs of wear. Revision of the cup should be performed at the same stage if signs of loosening and wear are noticed. The prosthetic head and the prosthesis neck should also be examined carefully to identify any signs of wear or damage that require revision. IPD without prosthetic head damage and stable, intact acetabular shell can be managed by only revising the polyethylene liner.

## Conclusions

IPD is a specific complication noted in dual-mobility-bearing THA. This mode of failure is mainly caused by the polyethylene liner wear resulting in failure of the captive mechanism and dissociation of the prosthetic head-polyethylene liner articulation. Iatrogenic IPD may result from closed reduction attempts involving dual-mobility-bearing THA. Post-manipulation radiographs showing eccentric head position within the metal cup and the “bubble sign” of the migrated polyethylene liner are diagnostic for IPD. If radiographs are not conclusive and IPD is suspected, a CT scan should be obtained. IPD requires operative intervention to revise the polyethylene liner and any other damaged or loose implant components.

## References

[1] Murray DG, Crown RS, Dickersin K (1995). Total hip replacement. JAMA.

[2] Marya SK, Thukral R, Singh C (2008). Prosthetic replacement in femoral neck fracture in the elderly: results and review of the literature. Indian J Orthop.

[3] Nho SJ, Kymes SM, Callaghan JJ, Felson DT (2013). The burden of hip osteoarthritis in the United States: epidemiologic and economic considerations. J Am Acad Orthop Surg.

[4] (2021). Finalised Patient Reported Outcome Measures (PROMs) in England for Hip & Knee Replacements, April 2018-March 2019. https://digital.nhs.uk/data-and-information/publications/statistical/patient-reported-outcome-measures-proms/finalised-hip--knee-replacements-april-2018---march-2019#.

[5] Kunutsor SK, Barrett MC, Beswick AD, Judge A, Blom AW, Wylde V, Whitehouse MR (2019). Risk factors for dislocation after primary total hip replacement: a systematic review and meta-analysis of 125 studies involving approximately five million hip replacements. Lancet Rheumatol.

[6] Prudhon JL, Ferreira A, Verdier R (2013). Dual mobility cup: dislocation rate and survivorship at ten years of follow-up. Int Orthop.

[7] Woolson ST, Rahimtoola ZO (1999). Risk factors for dislocation during the first 3 months after primary total hip replacement. J Arthroplasty.

[8] Kim Y, Morshed S, Joseph T, Bozic K, Ries MD (2006). Clinical impact of obesity on stability following revision total hip arthroplasty. Clin Orthop Relat Res.

[9] Padgett DE, Warashina H (2004). The unstable total hip replacement. Clin Orthop Relat Res.

[10] Patel PD, Potts A, Froimson MI (2007). The dislocating hip arthroplasty: prevention and treatment. J Arthroplasty.

[11] Miller LE, Gondusky JS, Kamath AF, Boettner F, Wright J, Bhattacharyya S (2018). Influence of surgical approach on complication risk in primary total hip arthroplasty. Acta Orthop.

[12] De Martino I, Triantafyllopoulos GK, Sculco PK, Sculco TP (2014). Dual mobility cups in total hip arthroplasty. World J Orthop.

[13] De Martino I, D'Apolito R, Waddell BS, McLawhorn AS, Sculco PK, Sculco TP (2017). Early intraprosthetic dislocation in dual-mobility implants: a systematic review. Arthroplast Today.

[14] Waddell BS, De Martino I, Sculco T, Sculco P (2016). Total hip arthroplasty dislocations are more complex than they appear: a case report of intraprosthetic dislocation of an anatomic dual-mobility implant after closed reduction. Ochsner J.

[15] Schirmers J, Horazdovsky R, Marston S (2015). Early intraprosthetic dislocation of dual-mobility total hip arthroplasty implant following attempted closed reduction: a case report. Reconstr Rev.

[16] Farizon F, de Lavison R, Azoulai JJ, Bousquet G (1998). Results with a cementless alumina-coated cup with dual mobility. A twelve-year follow-up study. Int Orthop.

[17] Philippot R, Camilleri JP, Boyer B, Adam P, Farizon F (2009). The use of a dual-articulation acetabular cup system to prevent dislocation after primary total hip arthroplasty: analysis of 384 cases at a mean follow-up of 15 years. Int Orthop.

[18] Lachiewicz PF, Watters TS (2012). The use of dual-mobility components in total hip arthroplasty. J Am Acad Orthop Surg.

[19] Heckmann N, Weitzman DS, Jaffri H, Berry DJ, Springer BD, Lieberman JR (2020). Trends in the use of dual mobility bearings in hip arthroplasty. Bone Joint J.

[20] Castiello E, Moghnie A, Tigani D, Affatato S (2021). Dual mobility cup in hip arthroplasty: an in-depth analysis of joint registries [In Press]. Artif Organs.

[21] Sariali E, Lazennec JY, Khiami F, Catonné Y (2009). Mathematical evaluation of jumping distance in total hip arthroplasty: influence of abduction angle, femoral head offset, and head diameter. Acta Orthop.

[22] Philippot R, Adam P, Farizon F, Fessy MH, Bousquet G (2006). [Survival of cementless dual mobility sockets: ten-year follow-up]. Rev Chir Orthop Reparatrice Appar Mot.

[23] Philippot R, Farizon F, Camilleri JP (2008). [Survival of dual mobility socket with a mean 17 years follow-up]. Rev Chir Orthop Reparatrice Appar Mot.

[24] Massin P, Orain V, Philippot R, Farizon F, Fessy MH (2012). Fixation failures of dual mobility cups: a mid-term study of 2601 hip replacements. Clin Orthop Relat Res.

[25] Vermersch T, Viste A, Desmarchelier R, Fessy MH (2015). Prospective longitudinal study of one hundred patients with total hip arthroplasty using a second-generation cementless dual-mobility cup. Int Orthop.

[26] Philippot R, Boyer B, Farizon F (2013). Intraprosthetic dislocation: a specific complication of the dual-mobility system. Clin Orthop Relat Res.

[27] Addona JL, Gu A, De Martino I, Malahias MA, Sculco TP, Sculco PK (2019). High rate of early intraprosthetic dislocations of dual mobility implants: a single surgeon series of primary and revision total hip replacements. J Arthroplasty.

[28] Swiontkowski M (2013). Concern about early intraprosthetic dislocation in dual-mobility implants. JBJS Case Connect.

[29] Koper M, Verdijk R, Bos K (2019). Asymptomatic intraprosthetic dual mobility cup dislocation with increased metal ion levels. Arthroplast Today.

[30] Fehring KA, Berry DJ (2016). Dissociation and intrapelvic entrapment of a dual-mobility polyethylene component. Clin Orthop Relat Res.

[31] Mohammed R, Cnudde P (2012). Severe metallosis owing to intraprosthetic dislocation in a failed dual-mobility cup primary total hip arthroplasty. J Arthroplasty.

